# Early Pseudoprogression following Chemoradiotherapy in Glioblastoma Patients: The Value of RANO Evaluation

**DOI:** 10.1155/2013/690585

**Published:** 2013-08-13

**Authors:** Paulo Linhares, Bruno Carvalho, Rita Figueiredo, Rui M. Reis, Rui Vaz

**Affiliations:** ^1^Department of Neurosurgery, Hospital de São João, Alameda Prof. Hernâni Monteiro, 4200–319 Porto, Portugal; ^2^Faculty of Medicine, University of Porto, Praça Gomes Teixeira, 4099-002 Porto, Portugal; ^3^Department of Neuroradiology, Hospital de São João, Alameda Prof. Hernâni Monteiro, 4200–319 Porto, Portugal; ^4^Life and Health Sciences Research Institute, School of Health Sciences, University of Minho, Campus de Gualtar, 4710-057 Braga, Portugal; ^5^Molecular Oncology Research Center, Barretos Cancer Hospital, Rua Antenor Duarte Villella, 1331 Bairro Dr. Paulo Prata, Barretos 14784-400, SP, Brazil

## Abstract

*Introduction*. The aim of this study was to determine the frequency of pseudoprogression in a cohort of glioblastoma (GBM) patients following radiotherapy/temozolomide (RT/TMZ) by comparing Macdonald criterial to Response Assessment in Neuro-Oncology (RANO) criteria. The impact on prognosis and survival analysis was also studied. *Materials and Methods*. All patients receiving RT/TMZ for newly diagnosed GBM from January 2005 to December 2009 were retrospectively evaluated, and demographic, clinical, radiographic, treatment, and survival data were reviewed. Updated RANO criteria were used for the evaluation of the pre-RT and post-RT MRI and compared to classic Macdonald criteria. Survival data was evaluated using the Kaplan-Meier and log-rank analysis. *Results and Discussion*. 70 patients were available for full radiological response assessment. Early progression was confirmed in 42 patients (60%) according to Macdonald criteria and 15 patients (21%) according to RANO criteria. Pseudoprogression was identified in 10 (23.8%) or 2 (13.3%) patients in Macdonald and RANO groups, respectively. Cumulative survival of pseudoprogression group was higher than that of true progression group and not statistically different from the non-progressive disease group. *Conclusion*. In this cohort, the frequency of pseudoprogression varied between 13% and 24%, being overdiagnosed by older Macdonald criteria, which emphasizes the importance of RANO criteria and new radiological biomarkers for correct response evaluation.

## 1. Introduction

The current standard of care for newly diagnosed glioblastoma (GBM) remains surgical resection followed by radiotherapy with concurrent temozolomide (RT/TMZ) and then maintenance temozolomide for at least six months [1]. The addition of chemotherapy introduced new questions regarding biological response of the tumor and the surrounding brain. Pseudoprogression is a phenomenon of subacute imaging changes in human glioma subsequent to radiochemotherapy suggestive of progression, with or without associated clinical sequelae, which resolve spontaneously without further therapy [2]. It was first reported by Hoffman et al. [3] in 1979 and fully described by de Wit et al. [4] in 2004. The underlying pathophysiological mechanisms are not fully understood [5]. Some authors suggest that it likely reflects an inflammatory tissue reaction after a local treatment secondary to vascular and oligodendroglial injury, whereas others associate it with an exaggerated response to effective therapy [2, 6, 7].

The incidence of pseudoprogression, time of occurrence, and its impact on prognosis remain poorly defined. Incidence has been reported to vary between 12% and 64% in different series, and this phenomenon commonly occurs in the first 3 months after radiochemotherapy, occasionally persisting for up to six months after treatment [2, 5] Imaging changes suggestive of pseudoprogression consist of an increase in gadolinium enhancement on T1-weighted images and peritumoral edema [5, 8]. Despite technical advances in perfusion magnetic resonance imaging (MRI) and positron emission tomography (PET), no imaging technique is yet validated for detection of pseudoprogression and has not been incorporated into the latest response assessment criteria [9–11].

Genetic and molecular changes have also been associated with pseudoprogression. Specifically p53 overexpression [12], O(6)-methylguanine-DNA methyltransferase (MGMT) promoter methylation status [13], Ki67 indices [14], and isocitrate dehydrogenase 1 (IDH1) mutation have been reported as potential biomarkers for detecting pseudoprogression [15]. 

The response of brain tumors is classically reported using Macdonald criteria based on the two-dimensional measurement of the enhancing area on CT or MRI, in conjunction with clinical assessment and corticosteroid dose [6]. However, this response evaluation has important limitations since posttreatment contrast enhancement in brain tumors is nonspecific and may not always be considered as a true surrogate of tumor response [16]. Differentiation between pseudoprogression and real progression is critical for making decisions on future therapy and for predicting the prognosis in clinical practice [17].

The purpose of this study was to clarify the frequency of pseudoprogression after radiochemotherapy in a cohort of Portuguese patients with glioblastoma, by comparing RANO with the classical Macdonald evaluation.

## 2. Materials and Methods

One hundred seventeen adult patients with newly diagnosed glioblastoma were treated between January 2005 and December 2009 with radiochemotherapy according to the Stupp protocol. Seventy patients were available for complete radiological response assessment. 

All patients were retrospectively evaluated, and demographic, clinical, radiographic, treatment, and survival data were reviewed. The inclusion criteria were age ≥18 years with histologically proved glioblastoma and the availability of MRI imaging less than 72 hours postoperatively and at each 3 months to define response. Response assessment was independently performed by a neuroradiologist (RF), according to RANO criteria [11]. These data were subsequently compared to Macdonald criteria evaluation [18].  Table 1 summarizes the response assessment criteria as adapted from Wen et al. [11].

Pseudoprogression was defined as a 25% increase in tumor size or any new lesion followed by stabilization (stable disease) or response (partial or complete), with no therapy modification, for at least 6 months after completing the concomitant phase of the RT/TMZ [19]. According to RANO criteria, progression within <12 weeks after completion of chemoradiotherapy was only defined if there was new enhancement outside of the radiation field (beyond the high-dose region or 80% isodose line) or if there was unequivocal evidence of viable tumor on histopathologic sampling. So, given the difficulty of differentiating true progression from pseudoprogression, clinical decline alone as well as an increase of contrast enhancement within the radiotherapy field was not considered sufficient for definition of progressive disease within the first 12 weeks after completion of concurrent chemoradiotherapy [11]. 

Demographic data are collected and summarized in Table 2.

Temozolomide was administered at a dose of 75 mg per m^2^ concurrent with daily radiotherapy and followed by 150–200 mg per m^2^ for 5 days every 28 days. Radiation was delivered in 2 Gy/fr, for a total of 60 Gy in 30 fractions. Patients were first categorized as having early progression if there was progression at the first follow-up scan 1 to 3 months after chemoradiotherapy and no early progression if there was stable or responsive disease. Patients with early progression were further subdivided in pseudoprogression and true progression according to further radiological progression.

### 2.1. Statistics

The primary endpoint was the frequency of pseudoprogression and its effect on prognosis. The secondary endpoints were overall survival and progression-free survival calculated from the date of surgery. Kaplan-Meier product-limit analysis was conducted and 95% confidence intervals were calculated. Log-rank test was used to detect statistically significant differences in survival distributions. Data of patients who have not progressed or died were right censored in our analysis. Multivariate survival analysis using a forward stepwise Cox regression model was used to determine independent predictors of survival. Borderline significant variables (*P* ≤ 0.1) were included in the final model. SPSS software version 19.0 was used for the statistical analysis.

## 3. Results

**Figure 1:**
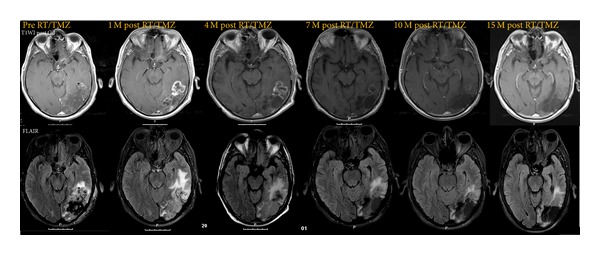
T1-contrast-enhanced and FLAIR magnetic resonance imaging (MRI) documenting pseudoprogression. Compared with pre-RT/TMZ MRI, the 1-month post-RT/TMZ showed increasing areas of contrast enhancement suggestive of tumoral progression. The patient remained clinically stable with stable dose of corticotherapy. Adjuvant TMZ treatment cycles were maintained and serial MRI at 4, 7, 10, and 15 months showed a consistent reduction in the size and the contrast of the lesion as well as in FLAIR signal.

At the first MRI scan performed 1 month after concurrent RT/TMZ, early progression was recorded in 42 patients (60%), while 28 patients were considered to have nonprogressive disease (nPD), according to Macdonald criteria. Thirty-four patients (81%) met the Macdonald criteria for progression due to a 25% increase in tumor size, 4 patients (9.5%) due to new lesions and 4 patients (9.5%) due to clinical deterioration. Out of the 42 patients with early progression there were 32 patients (76%) with further progression (tPD) and 10 (24%) patients with stable or reduced lesion—pseudoprogression (psPD) (Figure 1). Regarding nPD patients, 26 patients (93%) had stable disease and 2 patients (7%) showed partial response during subsequent follow-up.

When considering RANO criteria for progression within the first 12 weeks after completion of concurrent chemoradiotherapy, there were only 15 patients (21%) who met the criteria for progressive disease, while 55 patients (79%) were considered to have nonprogressive disease, either stable disease (27 patients), partial response (1 patient) or an increase of contrast enhancement within the radiotherapy field (27 patients), also considered stable disease. Out of the 15 early progressive patients, there were 14 patients with new enhancement areas outside of the radiation field and 1 patient with evidence of viable tumor on histopathologic sampling. Subsequently, 13 patients (87%) showed further progression (tPD) and 2 patients (13%) showed stable disease representing pseudoprogression (psPD).

 In fact, RANO criteria were specifically structured to make overcalling of early progression in the first 3 months difficult, so, as expected, progressive disease <12 weeks as assessed by RANO criteria revealed a lower value. 

**Figure 2:**
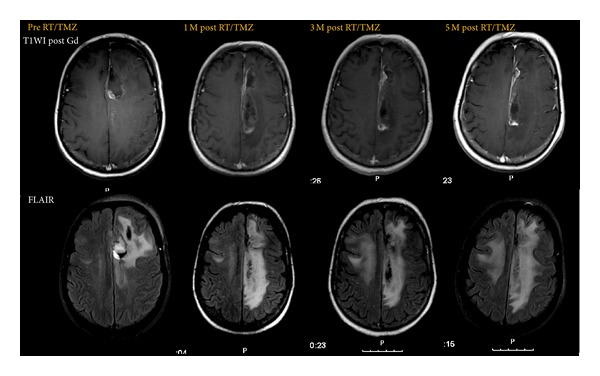
T1-contrast-enhanced magnetic resonance imaging (MRI) documenting pseudoprogression according to Macdonald criteria showing a significant increase in cingulate gyrus enhancing lesion at 1 month after RT/TMZ with subsequent stabilization (stable corticosteroid dose and functional status). FLAIR sequence shows increased signal extending to contralateral hemisphere anticipating true progressive disease.

After progressive disease in the first MRI 1 month after concurrent RT/TMZ, we observed dissociated responses between T2/FLAIR and T1-contrast-enhanced imaging in 3 patients in whom stabilization or decreasing enhancement was accompanied by an increase in T2/FLAIR signal without any modification in therapy (Figure 2). Subsequent imaging confirmed progression.

Regarding corticosteroid therapy in psPD group, 5 patients showed stable dose, 4 had decreasing doses, and only 1 had transient dose increment.

Functional status remained stable in patients with pseudoprogression according to the ECOG performance status.

### 3.1. Survival Analysis

The whole cohort median survival and median progression-free survival were 15 months [95% CI (13.64, 16.36)] and 10 months [95% CI (8.02, 11.98)], respectively. The only independent predictors of survival were the number of TMZ cycles and the type of surgery (Table 3). Interestingly, multivariate analysis showed that the number of TMZ cycles was a strong predictor for survival regardless of the ECOG status.

**Figure 3:**
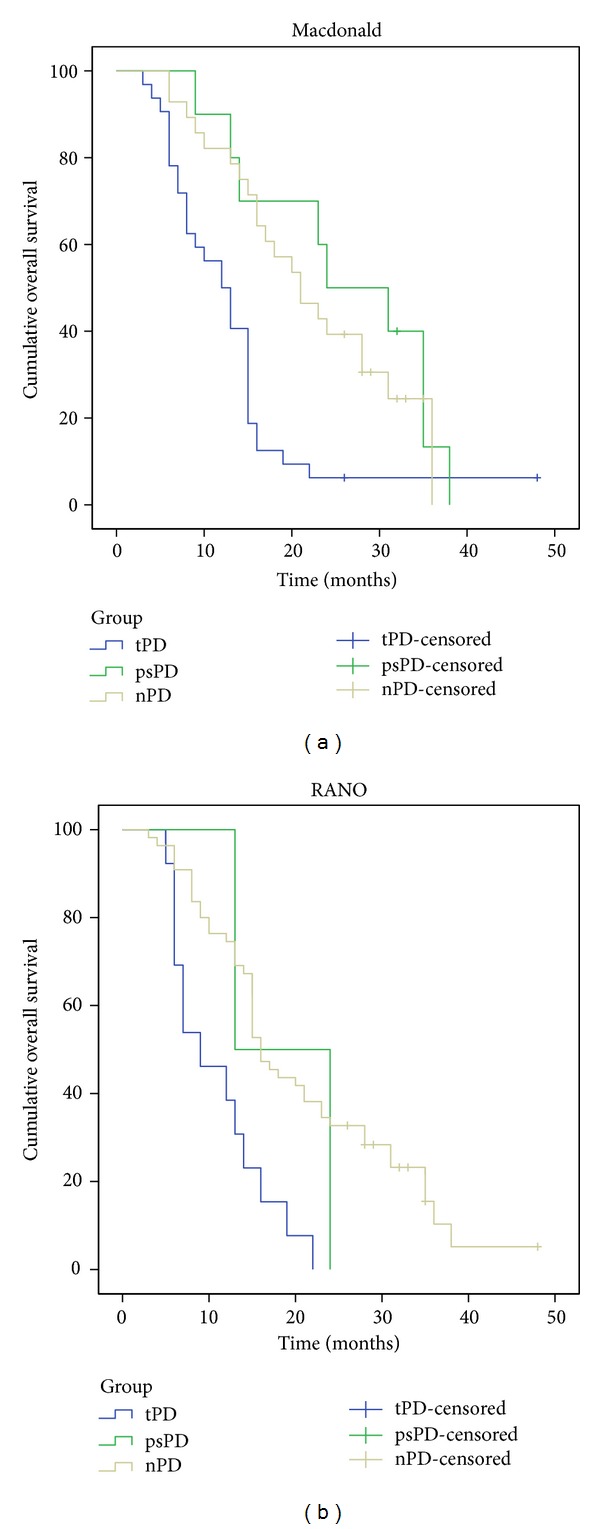
Kaplan-Meier analysis of *overall survival* curves according to different progression groups: true-progressive disease (tPD), blue; Pseudoprogression (psPD), green; Nonprogressive disease (nPD), yellow; and different response assessment criteria: (a), Macdonald; (b), RANO.

**Figure 4:**
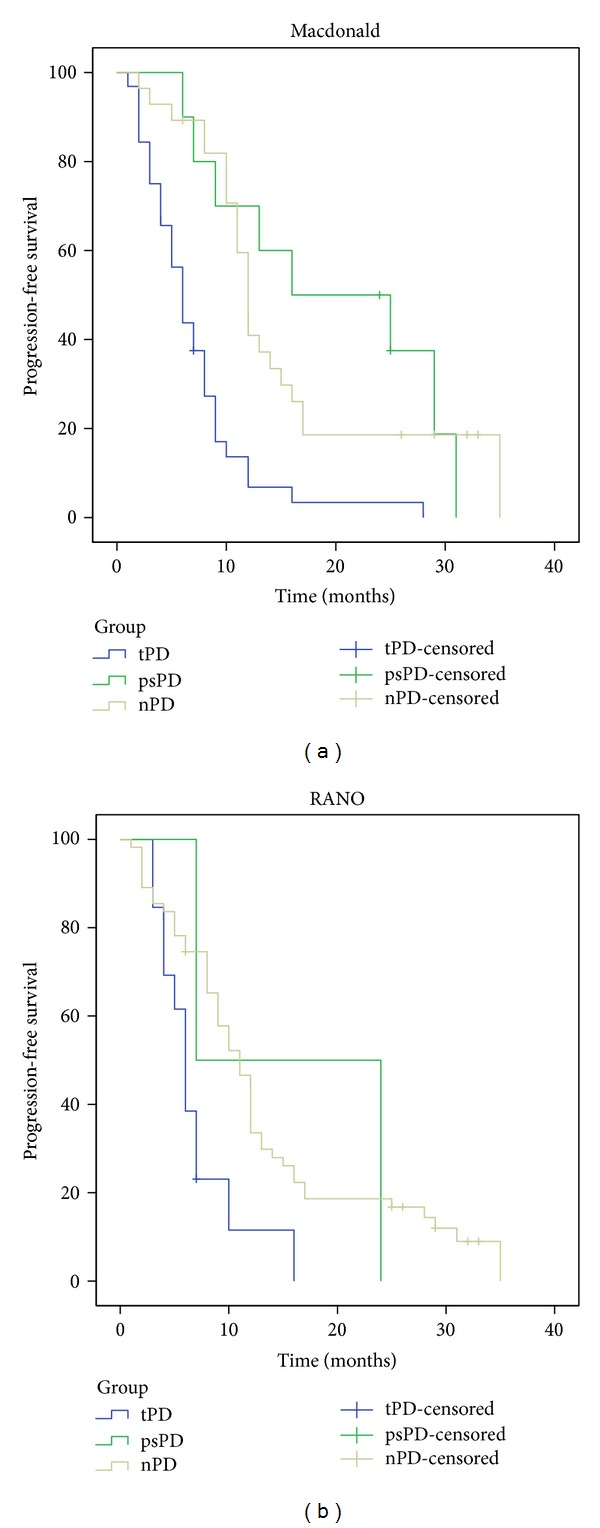
Kaplan-Meier analysis of *progression-free survival* curves according to different progression groups: true-progressive disease (tPD), blue; pseudoprogression (psPD), green; nonprogressive disease (nPD), yellow; and different response assessment criteria: (a) Macdonald and (b) RANO.

Survival analysis for subgroups of progression according to Macdonald criteria revealed a median overall survival of 12 months [95% CI (8.7, 15.3)] for the group of true progressive disease and 21 months [CI 95% (14.5, 27.5)] for the group of nonprogressive disease. Pseudoprogression group had a median overall survival of 24 months [CI 95% (11.6, 36.4)], not statistically different from the nonprogressive group (*P* = 0.456). According to RANO criteria, true progressive disease group had a median overall survival of 9 months [95% CI (3.7, 14.3)] and nonprogressive disease group had a median overall survival of 16 months [95% CI (13.8, 18.2)]. Pseudoprogression group was limited to 2 patients with an estimate median overall survival of 13 months, not statistically different from the nonprogressive group (*P* = 0.639) (Figure 3). Median progression-free survival for true progressive disease, pseudoprogression, and nonprogressive disease was 6 months, 16 months, and 12 months, respectively (Macdonald criteria applied) and 6 months, 7 months, and 11 months, respectively (RANO criteria applied) (Figure 4).

Comparing the survival distributions, patients with pseudoprogression showed a statistically significant improved overall survival over patients with true progressive disease (*P* = 0.01) and an increased time to progression (*P* < 0.001), according to Macdonald response assessment. Survival curves of psPD and nPD closely overlap showing no statistical difference (*P* = 0.456). Regarding RANO assessment group, the low rate of pseudoprogression (2/15 patients, 13%) did not provide enough statistical power to allow significant statistical conclusions in this particular group.

## 4. Discussion

Pseudoprogression is characteristically found within 2 to 3 months after treatment but can occur at 6 months and may progress over time into treatment-related necrosis [17, 31]. The issue of contrast enhancement areas starts even before adjuvant therapy administration. Farace et al. studied early MRI changes in the period between surgery and adjuvant therapy by diffusion-weighted imaging and MR perfusion, concluding that areas of new contrast enhancement of resected GBM are frequently observed in this period (17/37, 46%) and that most of them are suggestive of tumor progression (14/17; 82%) [32]. Distinguishing treatment induced imaging changes from true progressive disease can be especially difficult in the period immediately following completion of radiotherapy [8]. Standard MR imaging techniques do not allow a clear distinction between recurrent tumour and radiation-induced lesion [33]. The MRI characteristics of pseudoprogression have been described as an asymptomatic increase in contrast enhancement [34]. Enhancement of brain tumors primarily reflects a disturbed blood-brain barrier, and all the processes that decrease or increase this abnormal permeability will affect the area of enhancement, regardless of the size and activity of the tumor [31]. According to the literature, pseudoprogression occurs in 12 to 64% of patients.  Table 4, as adapted from Kruser et al., summarizes the data [5, 8, 10, 12–14, 17, 19–29]. 

Topkan et al. evaluated pathological confirmed incidence of pseudoprogression in a cohort of 63 patients. Pseudoprogression was identified in 12 out of 28 patients with early progressive disease (42.8%), representing an overall pseudoprogression rate of 19% [30]. The wide range of variation in retrospective cohorts might be explained by different criteria used to establish the response and, also, by considering different time periods. We only considered early progression in the first 3 months, and by this we could have lost some progressive patients. Our rates of 13 to 24% of pseudoprogression are in line with the literature. We retrospectively reviewed RANO criteria and compared them to Macdonald criteria since the latter do not take into account progressive nonenhancing disease, disregarding enlarging areas of nonenhancing tumor as evidence of tumor progression. Recently, a study comparing several response assessment criteria in recurrent GBM concluded a strong concordance between methods but noticed that criteria integrating FLAIR hyperintensity tended to reduce response rates and progression-free survival compared with criteria considering only contrast enhancement [35]. The decreased rate of pseudoprogression in our data after RANO evaluation reflects the improvement in response assessment. In fact, as discussed elsewhere, tumor progression as determined by both contrast-enhanced T1 and T2 sequences is more frequently diagnosed than when considering only contrast-enhanced T1 sequences [36]. Currently, changes in T2/FLAIR are not based on objective or volumetric measures due to technical limitations and insufficient standardization but should include mass effect and infiltration of the cortical ribbon or location outside of the radiation field [11].

The abnormal enhancement seen in pseudoprogression may be the result of treatment-related cellular hypoxia, which results in expression of hypoxia-regulated molecules from tumor and surrounding cells with the subsequent increased vascular permeability/increased tumor enhancement [7]. Pseudoprogression likely involves early changes to the vascular endothelium and the blood-brain-barrier [5]. It may be considered as a pathological continuum between acute post-RT reaction and treatment-related necrosis. More recently, Kruser et al. in their comprehensive review highlight the dichotomy between pseudoprogression—as a clinical diagnosis in the absence of intervention, and RT necrosis which can only be defined upon reoperation or biopsy [37]. Mechanisms behind these events are not fully understood but are probably related to microvessels changes. Post-RT brain injury induces a focal tissue reaction with inflammatory component and increased capillary permeability, causing fluid transudation into the interstitial space and brain oedema. Transient alterations in the blood-brain barrier may be responsible for new or increased contrast enhancement, falsely suggesting tumour progression, with low perfusion values [33].

There is also evidence that treatment-related necrosis occurs more frequently and earlier after temozolomide chemotherapy than after radiotherapy alone [31]. The MRI characteristics of pseudoprogression are similar to those of radiation necrosis, and conventional sequences are unable to distinguish between the two [28]. Corpus callosum involvement, subependymal spread, or multiple enhancing lesions are more consistent with progressive disease than pseudoprogression but are not able to reliably discriminate between both responses [28, 38]. Additionally, nonenhanced tumor is usually not considered in response evaluation as shown in the several retrospective studies. This represents a strong limitation in response assessment. Some advanced MRI techniques have shown promising results of distinguishing posttreatment necrosis from tumor progression and pseudoprogression, including MR spectroscopy (higher Cho/Cr and Cho/NAA ratios), MR perfusion imaging, and diffusion-weighted MRI, namely, relative cerebral blood volume (rCBV) as an imaging biomarker for increased neoangiogenesis and apparent diffusion coefficient (ADC) value as an imaging biomarker of increased cellular density [10, 17, 38–41]. 

Our data are in agreement with the literature as we have noticed an increase in overall survival and in time to progression in patients with pseudoprogression compared to patients with true progression as assessed by classical criteria. On the other hand, our results show no differences between the pseudoprogression patients and the patients with nonprogressive disease in contrast to some authors who report survival benefit in pseudoprogression patients [13] although the small number of patients enrolled in our study may preclude conclusions on the significance of this finding. RANO criteria assessment provided a very low rate of pseudoprogression patients due to the stringent nature of these criteria, especially within the first 12 weeks after chemoradiotherapy, as shown by the low rate of progressive disease during this period. This prevented definitive conclusions regarding the prognostic value of RANO criteria. Some authors reported that while incidence of pseudoprogression strongly depends on the applied criteria, the impact on survival remains the same, regardless of the stringency of the criteria [42]. The prognostic accuracy of different response assessment criteria is, however, yet to be proven in studies of greater magnitude. Nonetheless, the importance of making overcall of early progression difficult with RANO criteria cannot be overemphasized. Failure to exclude patients with pseudoprogression from recurrent gliomas trials with resulting falsely high responses rates and PFS demands the implementation of these strict criteria [11].

The data presented address the issue of the variability of pseudoprogression phenomenon when evaluated with conventional MRI. Novel reliable biochemical and imaging biomarkers are emerging and prospectively designed, and validated investigation is needed to conclude their clinical impact. 

## 5. Conclusions

Pseudoprogression is a phenomenon that appears after chemoradiation therapy for high-grade gliomas, and its pathophysiology is not fully understood. Rates vary widely and are dependent on the method of evaluation. The Macdonald criteria do not take into account all variables implicated in pseudoprogression and are being replaced. In this cohort of GBM patients, the frequency of pseudoprogression varied between 13% and 24%, being over-diagnosed by the older Macdonald criteria, which emphasizes the importance of RANO criteria and new radiological biomarkers for correct response evaluation. The accurate diagnosis of pseudoprogression is essential to avoid modifications of effective therapies or unnecessary treatments for GBM patients. More studies are needed to conclude the prognostic relevance of the different response assessment criteria.

## Figures and Tables

**Table 1:** Criteria for response assessment in high-grade gliomas.

Response	Criteria	
	Macdonald criteria	RANO criteria
Complete response	All: complete disappearance of all enhancing measurable and nonmeasurable diseases sustained for at least 4 weeks, no new lesions, no corticosteroids, and being stable or improved clinically.	All: T1 gadolinium enhancing disease, none; T2/FLAIR, stable or decreasing; new lesion, none; corticosteroids, none; clinical status: stable or improving.
Partial response	All: ≥50% decrease in sum of products of perpendicular diameters of all measurable enhancing lesions sustained for at least 4 weeks, no new lesions, stable or reduced corticosteroid dose, and being stable or improved clinically.	All: T1 gadolinium enhancing disease, ≥50% decrease; T2/FLAIR, stable or decreasing; new lesion, none; corticosteroids, stable or decreasing; clinical status: stable or improving.
Stable disease	All: being not qualified for complete response, partial response, or progression; being stable clinically.	All: T1 gadolinium enhancing disease: <50% decrease but <25% increase; T2/FLAIR: stable or decreasing; new lesion: none; corticosteroids: stable or decreasing; clinical status: stable or improving.
Progression	Any: ≥25% increase in sum of the products of perpendicular diameters of enhancing lesions, any new lesion, or clinical deterioration.	Any: T1 gadolinium enhancing disease: ≥25% increase; T2/FLAIR: increasing; new lesion: none; corticosteroids: not applicable; clinical status: deteriorating.

RANO: Response Assessment in Neuro-Oncology; FLAIR: fluid-attenuated inversion recovery.

**Table 2:** Demographic data of population.

Characteristics	Patients	
	Number	%
Age, years		
Median	62	
Range	34–78	
Gender		
Male	46	66
Female	24	34
ECOG		
0	18	25.7
1-2	43	61.4
3	9	12.9
Surgery		
Biopsy	14	20
Partial resection	32	46
Total resection	24	34
Adjuvant TMZ cycles		
Mean	5	
Range	0–20	
Corticotherapy		
Stable	43	61
Increased	11	16
Reduced	16	23

**Table 3:** Analysis of independent predictors of survival in the cohort of glioblastoma patients submitted to chemoradiotherapy.

Variable	Univariate analysis			Multivariate analysis		
	HR	95% CI	*P* value	HR	95% CI	*P* value
Age						
≥60 versus <60 years	1.26	0.75–2.10	0.389	—	—	—
Gender						
M versus F	0.98	0.57–1.69	0.943	—	—	—
Type of surgery						
Biopsy versus total or subtotal resection	**3.47**	**1.83**–**6.58**	**<0.001**	**2.60**	**1.35**–**4.99**	**0.004**
Number of surgeries						
1 versus >1	0.97	0.54–1.75	0.921	—	—	—
Corticosteroids						
Increasing versus stable or decreasing	1.04	0.54–2.02	0.903	—	—	—
ECOG						
0–2 versus >2	**0.34**	**0.17**–**0.72**	**0.005**	ns		
Number of TMZ cycles						
≥6 versus <6	**0.16**	**0.09**–**0.29**	**<0.001**	**0.18**	**0.10**–**0.32**	**<0.001**

CI: confidence interval; HR: hazard ratio; M: male; F: female; TMZ: temozolomide; ECOG: Eastern Cooperative Oncology Group; ns: nonsignificant. Results indicated in bold are significant. Multivariate analysis used Cox proportional hazards model with a forward stepwise regression procedure.

**Table 4:** Rates of pseudoprogression reported in the literature in patients treated with radiotherapy and temozolomide.

Author	Year	Number of patients	Response criteria	Period of early-response assessment (months)	Number with ePD	psPD (% of ePD)	Overall rate of psPD
Chamberlain et al. [20]	2007	51	Not specified	6	26	7/15 (47%)	7/40 (18%)
Brandes et al. [13]	2008	103	Macdonald	1	50	32/50 (64%)	32/103 (31%)
Taal et al. [19]	2008	68	Macdonald	1	31	15/31 (48%)	15/68 (22%)
Chaskis et al. [21]	2009	54	Gd-MRI + clinical	6	25	3/12 (12%)	3/54 (6%)
Clarke and Chang [22]	2009	85	Macdonald	0.5–1	35	10/27 (37%)	10/77 (13%)
Fabi et al. [23]	2009	12	Macdonald	2	4	2/4 (50%)	2/12 (17%)
Peca et al. [24]	2009	50	Gd-MRI + MR Spectroscopy	6	15	4/15 (27%)	4/50 (8%)
Roldan et al. [8]	2009	43	Macdonald	1–1.5	25	10/20 (50%)	10/38 (26%)
Gerstner et al. [25]	2009	45	Macdonald	0.5–1	24	13/24 (54%)	13/45 (29%)
Sanghera et al. [5]	2010	104	RECIST	2	27	7/22 (32%)	7/99 (7%)
Tsien et al. [10]	2010	27	Macdonald	3	14	6/14 (43%)	6/27 (22%)
Yaman et al. [26]	2010	67	Gd-MRI + T2/FLAIR	6	17	4/17 (24%)	4/67 (6%)
Gunjur et al. [27]	2011	68	Macdonald	1	41	14/41 (34%)	14/68 (21%)
Kang et al. [12]	2011	35	Macdonald	1	18	8/18 (44%)	8/35 (23%)
Kong et al. [17]	2011	90	Macdonald + rCBV	2	59	26/59 (44%)	26/90 (29%)
Young et al. [28]	2011	321	Macdonald	0.5–1	205	30/93 (32%)	Not specified
Park et al. [29]	2011	48	Macdonald	1	25	11/25 (44%)	11/48 (23%)
Topkan et al. [30]	2012	63	Macdonald	6	28	12/28 (43%)	12/63 (19%)
Pouleau et al. [14]	2012	63	Gd-MRI + edema	2	33	7/33 (21%)	7/63 (11%)
Present study	2013	70	RANO Macdonald	3	15 42	2/15 (13%) 10/42 (24%)	2/70 (3%) 10/70 (14%)

ePD: early progressive disease; tPD: true progressive disease; psPD: pseudoprogression; Gd-MRI: gadolinium enhanced-MRI.
